# Epigenetic Control of Haematopoietic Stem Cell Aging and Its Clinical Implications

**DOI:** 10.1155/2016/5797521

**Published:** 2015-11-22

**Authors:** Fizzah Aziz Choudry, Mattia Frontini

**Affiliations:** ^1^Department of Haematology, University of Cambridge, Cambridge CB2 0PT, UK; ^2^National Health Service Blood and Transplant, Cambridge Biomedical Campus, Cambridge CB2 0PT, UK; ^3^British Heart Foundation Centre of Excellence, University of Cambridge, Cambridge CB2 0QQ, UK

## Abstract

Aging, chronic inflammation, and environmental insults play an important role in a number of disease processes through alterations of the epigenome. In this review we explore how age-related changes in the epigenetic landscape can affect heterogeneity within the haematopoietic stem cell (HSC) compartment and the deriving clinical implications.

## 1. Introduction

Aging is associated with alterations in the heterogeneous haematopoietic stem cell (HSC) compartment including changes in clonal composition and lineage contribution. Recent data shows that these changes in functional potential within the HSC population may be modulated by a drift in the epigenome that occurs with increasing age and may ultimately lead to a transcriptional change within the HSC pool. Here we describe the current state of knowledge of haematopoietic stem cell heterogeneity and its changes with age, discuss the evidence for changes in the epigenetic landscape as a potential driver, and propose a model by which these changes may explain some of the pathological consequences of aging.

## 2. Haematopoietic Stem Cell Heterogeneity

The haematopoietic system relies on a small population of HSCs resident in the bone marrow to generate ~10^11^ new cells every day. HSCs have the capacity of self-renewal and differentiation through a cascade of progressively committed and lineage restricted progenitors to ultimately generate all mature circulating myeloid and lymphoid cell types [1]. Originally it was thought that HSCs were a single homogenous cell population with the same proliferation and multipotent differentiation capability [2]. However in the last 10 years it has become clear that the HSC compartment is in fact made up of a number of subsets each distinguished by its own self-renewal capacity (long-term and short-term HSCs) [3–5] and lineage differentiation potential [6].

The first evidence of HSC compartment heterogeneity came from mouse spleen CFU assays; these showed a high degree of variability in numbers and types of colonies produced, challenging the idea of a single homogenous population. However, direct evidence on HSC heterogeneity came from methods that allow assessment of mature cell outputs from limiting numbers and even single HSCs [7–10]. These include the ability of purified single HSCs to repopulate a secondary myeloablated host and cellular barcoding whereby lentiviral gene transfer is used to uniquely label individual HSCs allowing their progenies to be tracked within the transplanted host. This work has led to HSC subsets being distinguished according to their mature cell output. The existence of analogous HSC subsets in humans has been suggested by evidence from therapeutic transplantation in the setting of *β*-thalassemia [11].

Although currently defined HSC subsets are able to produce all the mature cell progeny, the ratio of myeloid to lymphoid progeny varies markedly. HSC compartment subsets have been described as myeloid-biased, lymphoid-biased, or balanced [3, 12] or by others as lymphoid-deficient (*α*), myeloid-deficient (*γ* and *δ*), and balanced (*β*), respectively [13] (Table 1). While the majority of accumulated evidence comes from mouse models there is also support for myeloid-biased and lymphoid-biased HSC subsets in humans [14, 15]. While serial transplantation experiments are able to define HSC subsets by their progeny of mature cells* in vivo* there is not yet an effective method to prospectively distinguish HSC subsets at molecular level. An attempt to prospectively enrich the myeloid- and lymphoid-biased subsets has been made by defining these populations based on CD150 cell surface level. Myeloid-biased HSC subsets express higher levels of this surface marker compared to lymphoid-biased HSCs [16–18]. However, this is not considered an ideal marker as the cell populations separated are not pure and expression of CD150 changes when cells are manipulated and transplanted [19, 20]. Recently lymphoid-biased HSCs have been shown to have higher expression of the surface marker CD229 [21].

A further HSC subset recently identified is the megakaryocytic or platelet-biased HSC subset (Table 1). This has been prospectively defined by reporter gene expression and/or surface markers that are highly expressed in the megakaryocyte/platelet lineage: VWF [22] and CD41 (ITGA2B) [23]. Approximately 60% of mouse HSCs have been shown to coexpress VWF and when these are serially transplanted in limiting numbers they effect highly platelet-biased reconstitution [22]. Interestingly, this population also has a strong myeloid lineage bias whereas VWF-HSC contribution to the myeloid lineage is minimal [22]. It has therefore been suggested that previous studies that identified a myeloid-biased HSC subset [3, 12, 13] may have identified both platelet-biased and myeloid-biased HSCs in the absence of methods to evaluate platelet output. Furthermore, the platelet-biased HSC subset has been hierarchically placed at the apex of the haematopoietic tree, due to its ability to give rise to the lymphoid-biased HSC subset [22]. Similarly, a platelet- and myeloid-biased HSC subset has been identified by the expression of the megakaryocyte/platelet cell surface marker and part of the glycoprotein IIb/IIIa fibrinogen receptor: CD41, a population that may possibly be phenotypically analogous to that expressing VWF [23]. CD41 had been known to be expressed in embryonic HSCs but then switched off after birth [24–27]. Its expression has now been demonstrated on a subset of mouse HSCs which show a platelet and myeloid bias on serial transplantation with a knockdown of CD41 resulting in reduced levels of all mature blood cell lineages [23]. A platelet-biased HSC subset has not yet been defined in the human.

While the majority of evidence on HSC lineage commitment to date, including that presented here, derives from transplantation studies, transplantation creates an artificial environment that is limited by engraftment-associated inflammation. The use of novel* in situ* inducible labelling techniques has enabled the study of physiological haematopoiesis in a healthy bone marrow environment. Two recent mouse studies using this approach have proposed a model of haematopoiesis that while supporting data from transplantation studies suggest that classical long-term HSCs have a limited contribution to steady-state haematopoiesis [28, 29]. Rather HSC heterogeneity is produced by thousands of multipotent clones within a reservoir of cells traditionally defined as short-term HSCs and multipotent progenitors [29], which are shown to be longer-lived than previously thought and have considerable self-renewal capability. These novel techniques while still in their early stages are anticipated to provide further insight into haematopoietic lineage commitment in more physiological conditions.

## 3. Aging within the HSC Compartment

Multiple studies have established that aging, both in mouse and in human, leads to a myeloid-skewed haematopoietic system, with diminished representation of lymphoid cell populations and an increased representation of myeloid progenitors that has been shown to be associated with a myeloid-biased HSC population [17, 30–32]. Furthermore, it has been shown that serial transplantation of young mouse HSCs into young secondary hosts selectively expands a myeloid-biased HSC population independent of a nonaging microenvironment which suggests that HSC lineage bias is intrinsic to the cell itself [32]. In addition, there is also evidence in the mouse of an age-related increase in platelet-biased HSCs defined by their expression of CD41. When transplanted these cells show a predominantly platelet- and myeloid-biased reconstitution, which suggests that the myeloid-biased HSC population seen in elderly mice may also be platelet-biased [23]. Although age-related changes in VWF+ HSCs have not been evaluated it could be speculated that this population would also expand with age since this subset leads to a platelet- and myeloid-biased reconstitution. This is supported by similarities in gene expression profiles between VWF+ HSCs and aged HSCs, both demonstrating significant upregulation of megakaryocyte-lineage genes such as* Selp* and* Clu* as well as the upregulation of* VWF* in aged HSCs [33]. Expansion of the VWF+ HSC population with age does not, however, support the hierarchical positioning of this population above the lymphoid-biased HSC subset as this population is known to decrease with age.

In humans, age-related haematopoietic changes include decreased bone marrow cellularity [34], attenuated lymphoid potential [35], increased incidence of myeloproliferative disorders and myeloid malignancies [36], and increased incidence of thrombosis [37]. As in mouse, these findings have been correlated with an accumulation of HSCs within the aged human bone marrow, which, while being able to generate both lymphoid and myeloid progeny in culture and in xenotransplant, showed significant myeloid skewing compared with young HSCs [14]. The existence of an age-related platelet-biased HSC subset in humans has not yet been investigated.

Postulated mechanisms that may lead to a decline in lymphoid differentiation with age include a gradual erosion of lymphopoietic potential within the HSC compartment over time, a conversion of lymphoid-biased to myeloid-biased HSCs, and a gradual dominance of myeloid-biased HSCs either due to their slower turnover leading to increased survival or a higher self-renewal capacity leading to clonal dominance with time [12, 38]. How these changes are controlled and regulated is still unclear. Aging within the HSC compartment has been shown to be associated with decreased functionality due to elevated levels of reactive oxygen species [39] and accumulation of DNA damage [40] which may account for some of the differences observed with age. It is clear, however, that these mechanisms do not account for all of the cellular and molecular attributes that are associated with aging of the HSC compartment, indicating that other mechanisms must be involved. There are a number of lines of evidence showing that HSC aging is transcriptionally regulated with differences in gene expression between young and aged HSC populations [14]. This suggests that alteration in gene expression by changes in the epigenetic landscape may play a key role in modulating age-related changes in the HSC compartment.

## 4. Epigenetic Regulation of HSC Aging

The term epigenetic encompasses all heritable changes in gene expression that are not due to changes in DNA sequence. These are modifications of the genome or of DNA-associated proteins. They include changes in DNA methylation, histone modifications, and changes in chromatin structure that impact on the accessibility of genetic loci for transcription machinery. Noncoding RNAs also play a critical role in epigenetic regulation. It is because of epigenetic regulation that a cell retains its identity and its gene expression profile through cell division and differentiation without altering its DNA sequence. However, epigenetic marks can also change over time [41–43] due to aging and environment, which may be related to mutations in epigenetic regulators, although the underlying molecular mechanisms are still unclear. It is this change in the epigenetic landscape of the HSC compartment that has been suggested to lead to age-related changes.

While the coding potential of the genome lies in the arrangement of the four nucleotides, additional information affecting phenotype is stored in the distribution of methylated cytosine (5-methylcytosine). DNA methylation occurs at CpG motifs that are interspersed within the genome in clusters called CpG islands. Densely methylated promoter regions are associated with compacted chromatin structure and therefore transcriptional shutdown; conversely demethylation leads to chromatin opening and therefore gene expression.

Aging in somatic tissues has been associated with global hypomethylation [44] where the majority of cells are postmitotic. In contrast in aged HSCs, which are characterised by mitotic potential often longer than the organism lifespan, a significant degree of global DNA hypermethylation is observed. However, if HSCs are taken to the end of their proliferation potential by serial transplantation, although not a physiological condition, similar patterns of global hypomethylation are observed [33, 45]. Young HSCs gain DNA methylation in regions associated with nonhaematopoietic lineages and significant losses of DNA methylation in genomic regions associated with blood cell production. Conversely, aged HSCs display gains of DNA methylation in genomic regions associated with lymphoid and erythroid lineages; both lineages decline in number during aging. Interestingly, the majority of genes differentially methylated during HSC aging were associated with lineage potential and highly expressed downstream of the HSC in the haematopoietic tree [45]. Furthermore, age-related hypermethylated regions were enriched for targets of the Polycomb group of proteins, known to establish repressive chromatin [33, 45].

Regulators of DNA methylation include DNA methyltransferases (DNMTs) that drive methylation of CpG motifs and the ten-eleven translocation (Tet) enzymes that regulate demethylation. Functional studies implicate these epigenetic regulators in the aging process within the HSC compartment. Genetic alteration studies demonstrate that DNMT1 is responsible for maintaining methylation and its loss in the HSC compartment leads to myeloid skewing and self-renewal defects [46, 47]. Furthermore, loss of both DNMT3A and DNMT3B leads to a severe arrest in HSC differentiation [48]. Loss of Tet2 in mice attenuates differentiation and leads to myeloid transformation and myeloid malignancies [49–51]. Somatic mutations in Tet2 have also been shown in normal elderly human subjects [52]. Importantly, there is now evidence of differential expression of both DNMT and Tet2 enzymes in aged HSCs compared with young HSCs [33, 45].

Histone posttranslational modifications including acetylation, methylation, phosphorylation, sumoylation, and ubiquitination can change chromatin structure and therefore DNA accessibility to transcriptional machinery. These modifications may act separately or synergistically to regulate gene expression. Priming DNA in such a way precedes lineage commitment in the HSC population as seen by histone modifications associated with gene expression in committed mature cell populations already present within the HSC compartment [53, 54]. This observation is similar to that of differential DNA methylation of genes that are expressed downstream described above. HSCs also utilise Polycomb (PcG) genes to regulate aging, prevent premature aging, and maintain HSC function by forming PcG repressive complexes (PRC). While the PRC1 complex possesses H2AK119 ubiquitin ligase activity, PRC2 acts as a H3K27 methyltransferase.

Age-related changes in histone modifications also provide mechanisms that may contribute to changes seen in the aged HSC compartment. Aged HSCs show methylation of H3K4me3, a mark of active chromatin, which correlate with increases in gene expression of HSC identity and self-renewal genes [33]. Differential methylation of the repressive mark H3K27me3 has also been shown in aged HSCs, with increased H3K27me3 on a number of promoters [33]. HSC aging is also associated with low levels of H4K16ac activation mark [55]. Furthermore, interdependency between DNA methylation and histone modification exists and it might be relevant to HSC aging; however its full understanding is reliant on the development of assays that require smaller cell numbers to detect both epigenetic marks in the same samples.

Functional studies, where lysine-specific demethylases that drive H3K4 demethylation and regulate chromatin accessibility have been genetically modulated, show their critical role in stem cell differentiation. Kdm3a and Kdm5a have also been implicated in regulating stem cell aging, a notion supported by the fact that these proteins' expression decreases with age [33, 56, 57]. Knockdown of the lysine demethylase Kdm5b (Jarid1b) leads to increased HSC activity [58] and is also known to be differentially expressed with aging [45]. Knockout studies of the H3K27me3 demethylase Kdm6a (UTX1) have shown it to be a key regulator of haematopoiesis [59] and knockdown in* C. elegans* extends their lifespan [60]. Furthermore, HSCs deficient in the Bmi1 component of PRC1 [61–63] as well as the Ezh1 [64] and Eed [65] components of PRC2 show a severe defect with marked derepression of the tumour suppressor and aging-associated complex Ink4a/Arf.

Noncoding RNAs are RNAs that are not translated into protein but are known to play an important epigenetic regulatory role. While the direct impact of noncoding RNAs on HSC aging requires further investigation, there is evidence to suggest that noncoding RNAs are both highly expressed and regulate HSC survival and function. The microRNA miR-125b is highly expressed in HSCs and plays a role in regulating survival [66, 67] whereas miR-126 knockdown is associated with a myeloid-biased HSC compartment [68]. The long noncoding RNA Xist is essential for HSC survival [69].

## 5. Consequences of Age-Related Changes in the HSC Epigenetic Landscape

**Figure 1:**
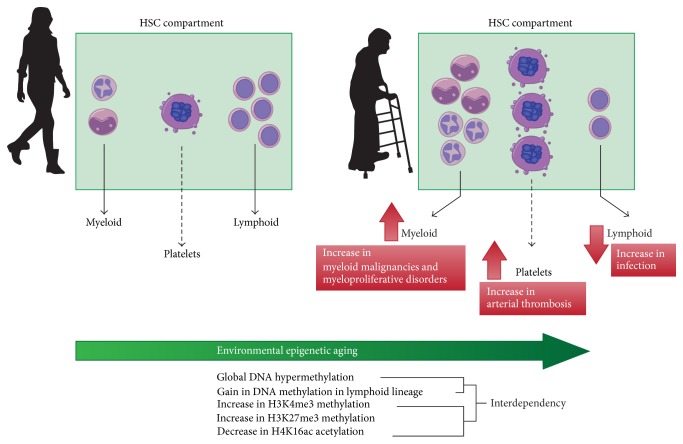
Drift in the epigenetic landscape that occurs with environmental and biological factors associated with aging leads to transcriptional differences between the HSC compartments in the young population compared with the elderly population. This is proposed to give rise to expansion of particular clones within the heterogeneous HSC pool to produce a myeloid- and platelet-skewed haematopoietic system. These changes may play an important role in driving the increased incidence of myeloproliferative disorders, myeloid malignancies, infection, and acute arterial thrombosis observed in the elderly.

It is not clear if there is a physiological benefit of a progressive myeloid bias within the HSC compartment. However, it is clear that the epigenetic drift that leads to this phenotype correlates with the increased incidence with age of myeloproliferative disorders and myeloid malignancies as well as increased risk of infection and thrombosis (Figure 1).

Myeloproliferative disorders, myelodysplastic syndromes, and haematological malignancies [36] are attributed to accumulation of mutations in the aging HSC compartment, many of which are involved in epigenetic regulation of the HSC population such as Tet2 and DNMT3 [52, 70–73]. DNA methylation plays an important role in the pathogenesis and progression of myelodysplastic syndromes where DNA hypermethylation and methyl silencing are implicated as the pivotal mechanism [74–76]. In accordance with this, clinical trials for agents that inhibit DNA methylation are ongoing in myelodysplastic syndromes [77–80]. Acute myeloid leukaemia is typically associated with hypomethylation caused by deregulation of DNMT1 or possibly overexpression of Tet family genes [81, 82].

The myeloid skewing that occurs with advancing age may also be associated with the decline in adaptive immune response with a resultant increased risk of infection that confers high levels of morbidity and mortality in the elderly population. There is a known reduction in numbers of circulating naïve lymphocytes that could be a direct effect of a changing epigenetic landscape within the HSC compartment leading to a reduced mature lymphoid lineage generation [35, 83–85].

Age is also an important risk factor for coronary and cerebrovascular platelet thrombosis [37], conditions that have been causally linked to increased platelet activity and increased platelet mass [86]. The setting of acute arterial thrombosis has been likened to states of increased haemostatic demand. While only correlative and chronological data exists, it is reasonable to hypothesise that as in states of increased haemostatic demand, when functional circulating platelet mass drops, there is a consequential regulation of megakaryocyte activity and platelet production. The nature of this control system and how megakaryocyte activity is regulated is still unknown; however, increases in platelet mass have been correlated with increased megakaryocyte ploidy [87–89]. It is also possible that this feedback may be at the level of the HSC by an expansion of a platelet-biased HSC subset. With an age-related increase in both platelet mass [90, 91] and platelet-biased HSCs [23] it could be suggested that the recently discovered megakaryocyte- or platelet-primed HSC subset may have thrombotic implications in the elderly. It is reasonable to hypothesise that age-related changes in the epigenetic landscape with age lead to a platelet-biased HSC subset that is primed to generate a transcriptionally distinct megakaryocyte phenotype due to defined epigenetic marks that leads to increased platelet mass and activity. In support of this increasing megakaryocyte ploidy is associated with significantly increased expression of VWF and CD41 [92], both markers of a platelet-biased HSC subset.

A complex relationship exists between megakaryocytes and HSCs, with a number of phenotypic and molecular similarities including surface markers (CD41 and VWF), thrombopoietin (TPO), its receptor (MPL) and CXCR4, transcription factor dependence (RUNX-1, GATA-2, Evi-1, SCL/TAL-1, and Ets family transcription factors), signalling pathways, and proximity within the bone marrow niche [93]. Furthermore, maintenance of both megakaryocytes and HSCs crucially depends on TPO [94–96]. While the functional relevance of these similarities remains unclear there is now a body of evidence that supports the existence of tight homeostatic control mechanisms along the HSC-megakaryocyte-platelet axis. Recent reports demonstrate a critical role for megakaryocytes in maintaining HSC quiescence through either release of CXCL4 [97] and TGF*β* [98] or indeed CD41 expression [23]. In mouse acute depletion of megakaryocytes leads to HSC expansion and proliferation implying a critical regulatory feedback mechanism between megakaryocytes and the HSC compartment [97]. Moreover, platelets may also regulate HSC quiescence through effects on circulating TPO concentrations [99]. Further work is required to gain a full understanding of this complex relationship.

## 6. Conclusions

Epigenetic changes in the HSC compartment lead to the phenotypic and functional changes that are seen in the mature cell output of the haematopoietic system with advancing age. Although these epigenetic changes are not directly pathological they produce an environment that is conducive to pathological processes that are seen in prevalence in the elderly population. In some disease processes age-related epigenetic changes may be more directly pathogenic but in other complex diseases such as coronary artery disease they may indeed account for the missing heritability determinants that have not been accounted for to date by genetic studies of sequence variation [100]. Our understanding of the role of epigenetic changes in stem cell regulation, though quickly expanding, is only at its beginnings. However, as epigenetic marks are potentially reversible this opens up the possibility of manipulating epigenetic states and ultimately changing the way the genome functions.

## Figures and Tables

**Table 1:** Currently defined HSC subsets in mouse and their definition by mature cell output and cell surface markers.

HSC subset	Predominant mature cell population	Cell surface markers that have been used for prospective identification
*Mouse HSC *		Lin^−^ Sca-1^+^ cKit^+^ CD150^+^ CD48^−^ CD34^−^
Myeloid-biased/lymphoid-deficient (*α*)	Myeloid	+CD150^High^, CD41^+^
Lymphoid-biased/myeloid-deficient (*γ*/*δ*)	Lymphoid	+CD229^+^
Balanced (*β*)	Myeloid and lymphoid	
Platelet-biased	Platelet and myeloid	+VWF^+^, CD41^+^
*Human HSC *		Lin^−^ CD34^+^ CD38^−^ CD90^+^ CD45RA^−^ CD49f^+^

Lin: lineage markers; VWF: von Willebrand factor.
